# Treatment and Valorization of Waste Wind Turbines: Component Identification and Analysis

**DOI:** 10.3390/ma18020468

**Published:** 2025-01-20

**Authors:** Xiaohan Zhao, Daria Pakuła, Miłosz Frydrych, Roksana Konieczna, Bogna Sztorch, Rafał Kozera, Hongzhi Liu, Hui Zhou, Robert E. Przekop

**Affiliations:** 1 International Center for Interdisciplinary Research and Innovation of Silsesquioxane Science, Key Laboratory of Special Functional Aggregated Materials, Ministry of Education, School of Chemistry and Chemical Engineering, Shandong University, Jinan 250100, China; 202320367@mail.sdu.edu.cn (X.Z.); liuhongzhi@sdu.edu.cn (H.L.); 2 Center for Advanced Technologies, Adam Mickiewicz University in Poznań, Uniwersytetu Poznańskiego 10, 61-614 Poznań, Poland; darpak@amu.edu.pl (D.P.); frydrych@amu.edu.pl (M.F.); rokkon@amu.edu.pl (R.K.); bogna.sztorch@amu.edu.pl (B.S.); 3 Faculty of Chemistry, Adam Mickiewicz University in Poznań, Uniwersytetu Poznańskiego 8, 61-614 Poznań, Poland; 4 Faculty of Materials Science and Engineering, Warsaw University of Technology, ul. Woloska 141, 02-507 Warszawa, Poland; rafal.kozera@pw.edu.pl; 5 Key Laboratory for Thermal Science and Power Engineering of Ministry of Education, Beijing Key Laboratory of CO2 Utilization and Reduction Technology, Department of Energy and Power Engineering, Tsinghua University, Beijing 100084, China; huizhou@tsinghua.edu.cn

**Keywords:** wind turbines, waste composite, recycling, epoxy resin, polyester resin

## Abstract

Recycling end-of-life wind turbines poses a significant challenge due to the increasing number of turbines going out of use. After many years of operation, turbines lose their functional properties, generating a substantial amount of composite waste that requires efficient and environmentally friendly processing methods. Wind turbine blades, in particular, are a problematic component in the recycling process due to their complex material composition. They are primarily made of composites containing glass and carbon fibers embedded in polymer matrices such as epoxies and polyester resins. This study presents an innovative approach to analyzing and valorizing these composite wastes. The research methodology incorporates integrated processing and analysis techniques, including mechanical waste treatment using a novel compression milling process, instead of traditional knife mills, which reduces wear on the milling tools. Based on the differences in the structure and colors of the materials, 15 different kinds of samples named WT1-WT15 were distinguished from crushed wind turbines, enabling a detailed analysis of their physicochemical properties and the identification of the constituent components. Fourier transform infrared spectroscopy (FTIR) identified key functional groups, confirming the presence of thermoplastic polymers (PET, PE, and PP), epoxy and polyester resins, wood, and fillers such as glass fibers. Thermogravimetric analysis (TGA) provided insights into thermal stability, degradation behavior, and the heterogeneity of the samples, indicating a mix of organic and inorganic constituents. Differential scanning calorimetry (DSC) further characterized phase transitions in polymers, revealing variations in thermal properties among samples. The fractionation process was carried out using both wet and dry methods, allowing for a more effective separation of components. Based on the wet separation process, three fractions—GF1, GF2, and GF3—along with other components were obtained. For instance, in the case of the GF1 < 40 µm fraction, thermogravimetric analysis (TGA) revealed that the residual mass is as high as 89.7%, indicating a predominance of glass fibers. This result highlights the effectiveness of the proposed methods in facilitating the efficient recovery of high-value materials.

## 1. Introduction

Wind energy is considered one of the cleanest technologies for generating electricity, with low environmental impact during operation [1,2]. Wind energy contributes to reducing greenhouse gas emissions, and the operation of turbines is associated with minimal air and soil pollution [3,4]. The year 2023 marked the highest annual installed capacity of onshore wind power, exceeding 100 GW for the first time in a single year, reaching 106 GW, representing a year-on-year growth of 54% [5]. Additionally, 2023 represented the second-best year for offshore wind capacity, totaling 10.8 GW. The world’s cumulative installed wind capacity surpassed the one-terawatt (TW) milestone, with the total reaching 1021 GW, representing a 13% year-on-year increase. The top five global markets were China, the United States, Brazil, Germany, and India. China contributed a record 75 GW of new installed capacity, constituting nearly 65% of the global total [6,7]. The wind power industry is entering a new era of accelerated growth. China’s wind power industry has a promising future and will play an increasingly important role in the global energy transformation, according to the latest report from China’s National Energy Administration. As of the end of September 2024, China’s installed wind power capacity reached approximately 480 GW, representing a year-on-year increase of 19.8%. China aims to expand its installed wind power capacity to at least 1200 GW by 2030 [8]. The development of offshore wind power is also supported by national policies, and it is expected that by 2025, the lifting capacity of offshore wind power can reach 12 GW.

Wind turbines are generally classified into the following categories based on their power output: small wind turbines (<1 MW) with blade diameters generally less than 20 m; medium-sized wind turbines (1–3 MW) with blade diameters ranging from approximately 30 to 50 m; large wind turbines (3–6 MW) featuring blade diameters of up to 60 to 120 m; and giant turbines (>6 MW) with blade diameters exceeding 150 m [9]. For instance, China is actively advancing the adoption of giant offshore wind turbines with capacities of 10 MW and above to enhance the efficiency and cost-effectiveness of offshore wind power [10].

However, after years of use, turbines lose their functional properties, generating significant amounts of composite waste [11,12,13,14]. Managing this waste requires effective and environmentally friendly processing methods, as these materials, particularly the blades, are difficult to recycle due to their complex structure and the use of high-strength materials [15,16,17,18,19]. The recycling of wind turbines that have reached the end of their life cycle presents an increasing challenge [19]. The number of decommissioned turbines is steadily rising with the growth of the global wind energy sector, which, in turn, leads to an increase in the amount of waste that must be properly processed. This stage has been identified as a critical weak point in life cycle analyses of wind turbines, as past research has focused primarily on the operational, production, and installation phases [20]. More practical experience is needed in efficiently dismantling and reusing materials derived from wind turbines. In response to these challenges, a growing body of research focuses on developing technologies for processing wind turbine waste, particularly the blades, which are among the most difficult components to recycle.

The complex composites of wind turbine blades are fibers, matrix, and sizing. Currently, fibers mainly contain glass and carbon fibers, aramid and basalt fibers, hybrid composites, and natural fibers. Typically, the glass/epoxy composites for wind blades contain up to 75 weight % of glass fibers [21]. Carbon fiber is considered a very promising alternative, but is also not widely used due to its high cost and limited properties [22]; aramid and basalt fibers, hybrid composites, and natural fibers are also considered interesting substitutes [22]. Thermosets (epoxies, polyesters, and vinyl esthers) or (more seldom) thermoplastics are used as matrices in wind blade composites. For sizing, an organosilane, chromium, or titanium oxides are often chosen. Organosilanes can react with the glass fiber surface through a sol-gel reaction, which can covalently bond the organosilane or a polymeric form of the organosilane to the fiber surface. The complexity of components makes it difficult to recover, and it is a great challenge to find a scientific and effective method for recycling. Table 1 presents the turbine parts along with the materials used for their production.

There are many current recycling methods, such as mechanical, chemical, and thermal recycling [36,37,38,39]. Mechanical recycling uses more complex and advanced processes that offer the possibility of multiple industrial applications. The waste product can only be chopped as a filler, which is currently used in cement [40] where its use is limited. Traditional recycling methods often rely on equipment such as cutters, which frequently lead to high wear rates, increased energy consumption, and inefficient material processing. Furthermore, the lower quality of materials recovered through mechanical recycling limits their reuse potential, further raising the economic costs and environmental burden of the recycling process. Chemical recycling is another emerging technology that breaks down composites into their basic components, such as fibers and resins, but it is still in the exploratory stage [34]. As for thermal recycling, it is not a good solution due to its environmental impact, including emissions and energy consumption [34,41]. The overall application of turbine blades is also underway, which requires only cutting the blade and using it as a whole, with limited application scenarios [42]. These explorations have proven the importance of realizing the recycling of wind turbine blades, but there is still work to be carried out on how to reuse them in a viable way.

In this work, an innovative new compression milling process was used to mechanically treat the waste, using wet and dry methods to make the separation more efficient, in addition to several analytical tools, such as Fourier transform infrared spectroscopy (FTIR), thermogravimetric analysis (TGA), and differential scanning calorimetry (DSC), and the resulting particulate fractions were analyzed statistically and geometrically. This method can not only reduce the damage to the glass fiber by using the new machine grinding, but also the wet separation method can reduce the damage of small particles to the human respiratory system.

## 2. Materials and Methods

### 2.1. Preparation of Samples

**Figure 1:**
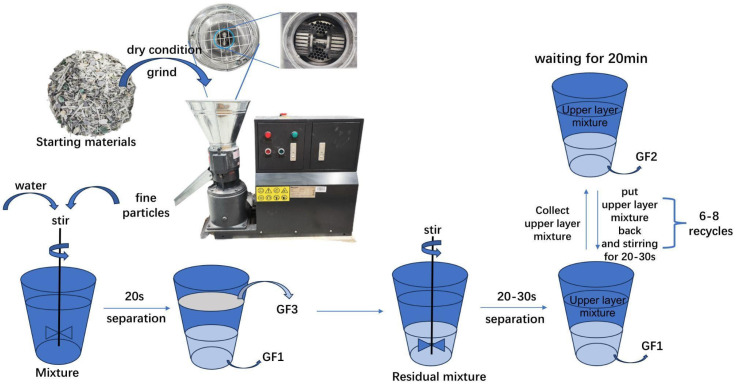
Separation method.

The wind turbine waste materials used in this study were provided by Anmet, a recycling company based in Szprotawa, Poland. The wind turbines originated from the Saxony region in Germany, ensuring the samples represent materials commonly used in European wind energy infrastructure, the starting materials are shown in the Figure 1.

#### 2.1.1. Manual Separation

Based on visual differences, the starting materials were sorted and labeled, all of the selected samples were presented in Table 2, then subjected to analyses such as FTIR, DSC, and TGA.

#### 2.1.2. Compression Milling Process

The remaining components of the starting materials, except large parts of resin and metals, were ground into fine particles. As shown in Figure 1, the novel compression milling process is used in this procedure as a substitute for conventional milling. The name of the equipment used in the process is Pellet Machine, brand name Wiesenfield, model number WIE-PM-1500. By increasing the compressive force and cutting speed during milling, this approach effectively reduces tool wear and enhances machining efficiency.

#### 2.1.3. Wet Separation

Water was added to the mixture and the upper layer light portion, named GF3, was collected after about 20 s of stirring; the remaining portion continued to be stirred for about 20–30 s to obtain the mixture and the heavier layer of GF1. The upper mixed layer was poured out the and left to stand for about 20 min to collect the GF2, then the upper layer was poured back into the mixture containing the GF1 and stirred for 20–30 s. The process was repeated 6–8 times to collect the final GF1 and GF2. Figure 1 illustrates the separation process.

#### 2.1.4. Dry Separation

The separated particles were placed in the dryer overnight at 60 °C. After drying, a screening machine with mesh sizes of 40 µm, 90 µm, 250 µm, and 1000 µm was used to sort the components of GF1 and GF2 into different size fractions. As there was only a small portion of GF3, it was separated using a small screening machine with sieves of 36 µm, 100 µm, 250 µm, and 1000 µm.

### 2.2. Analytical Methods

Fourier transform infrared (FTIR) spectra were recorded on a Nicolet iS50 Fourier transform spectrophotometer (Thermo Fischer Scientific, Waltham, MA, USA) equipped with a diamond ATR unit with a resolution of 0.09 cm^−1^.

Thermogravimetry (TGA) was performed using a NETZSCH 209 F1 Libra gravimetric analyzer (Selb, Germany). Samples of 8 mg ± 0.5 mg were cut and placed in Al_2_O_3_ crucibles. Measurements were conducted under nitrogen (flow of 20 mL/min) in temperature ranges from 30 °C to 1000 °C and at a 10 °C/min heating rate.

Differential scanning calorimetry (DSC) was performed using a NETZSCH 204 F1 Phoenix calorimeter (Selb, Germany). Samples of 6 ± 0.2 mg were placed in an aluminum crucible with a punctured lid. The measurements were performed under nitrogen in temperature ranges from 20 to 300 °C for all samples (except WT4, where the range was 20–200 °C) and at a 10 °C/min heating rate.

The surface structure was analyzed under a Digital Light Microscope Keyence VHX 7000 with 100× to 1000× VH-Z100T lens (Osaka, Japan). All of the pictures were recorded with a VHX 7020 camera.

## 3. Results

### 3.1. Visual Assessment

Based on the differences in the structure and the colors of the materials, samples were selected from crushed wind turbines, enabling a detailed analysis of their physicochemical properties and the identification of the constituent components. During the selection process, particular attention was paid to the color variations, which may indicate the presence of different types of plastics and additives used in the construction of the turbines. This diversity may result from employing various material formulations, highlighting the complexity of the composites utilized in the construction of wind turbines. The samples underwent further analyses, including optical microscopy, Fourier transform infrared spectroscopy (FTIR), and thermal studies, such as thermogravimetric analysis (TGA) and differential scanning calorimetry (DSC), to assess their morphology and chemical composition. The analyses allowed for the determination of the composition of the wind turbines, which are characterized by complexity due to the use of various components, such as glass fibers, polyester and epoxy resins, and other composite materials. Table 2 presents the selected samples, illustrating their diversity.

#### 3.1.1. Optical Microscopic Analysis

The surface structure analysis of selected materials, conducted using an optical microscope at three magnifications (×100, ×200, ×500), revealed diverse morphologies (Table 3). These variations result from mechanical processes and the heterogeneity of components constituting the wind turbines. The microscopic images of the WT1 sample reveal slight irregularities and microcracks. Dispersed micropores are observed, along with the presence of glass fibers. Sample WT3, identified as blue polyethylene, demonstrated a more porous structure with numerous irregularities. The surface of this sample appeared degraded, possibly due to external factors or mechanical processing. At higher magnifications, pores were visible, indicating structural changes typical for polymers exposed to demanding conditions. The WT4 sample under the microscope shows a dense, irregularly arranged fibrous/spongy structure with impurities, probably resins and glass fibers. The color tones vary in shading, suggesting a mixture of different components or phases within the material. Sample WT2 exhibited an uneven, rough surface, suggesting a complex structure with possible traces of mechanical processing. In contrast, samples WT5, WT6, WT8, and WT10-12 displayed a more uniform, linear morphology with regular micro-scratches, which could be attributed to the presence of glass fibers within the polymer matrix. Sample WT7, which is a metal, exhibited a surface with visible scratches. The microscopic images showed a regular structure, typical of metal, though defects such as scratches were observed, which could result from wear and mechanical processing. The metal showed greater resistance to surface damage compared to polymeric materials. Sample WT9 displays a metallic sheen surface, indicating that it is a metal fragment. The microscopic images of samples WT13, WT14, and WT15 reveal distinct material composition and the presence of phases with differing optical properties. The observed color contrasts and visible transitions between phases suggest that each sample may contain various components or additives. Microscopic images revealed numerous cracks and surface defects, characteristic of materials subjected to prolonged exploitation. Intense degradation was evident, which may result from exposure to moisture or chemicals, significantly weakening the metal’s structure.

#### 3.1.2. FTIR Analysis

**Figure 2:**
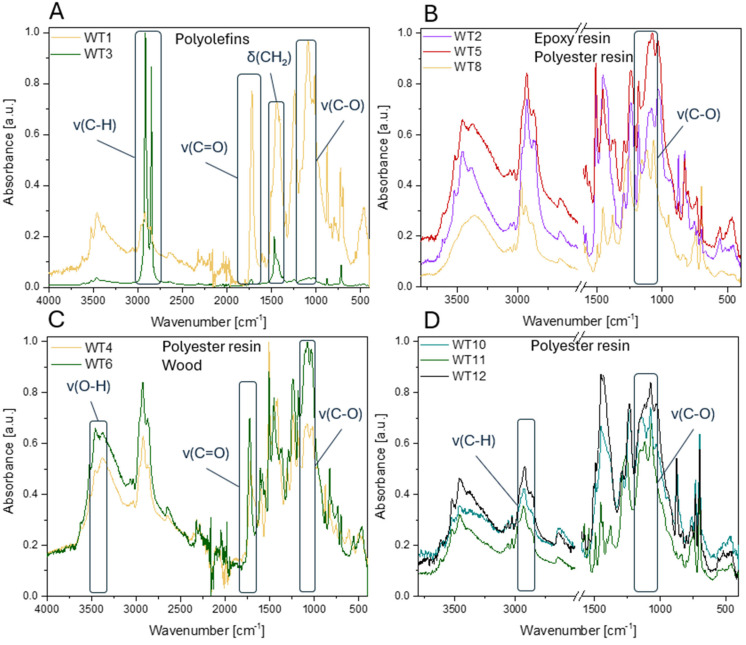
FTIR spectra of: (**A**) WT1, WT3; (**B**) WT2, WT5, WT8; (**C**) WT4, WT6; (**D**) WT10-12.

Each analyzed sample may contain a mixture of chemical compounds due to their complex nature as composite materials. This analysis aims to identify the dominant chemical compound. In the first stage of the analysis, the obtained FTIR spectra were compared with a reference database to aid in the identification of fundamental components. This comparative approach served as the foundation for subsequent detailed spectral analysis, which focused on identifying and interpreting characteristic signals, thereby enabling a more comprehensive and accurate interpretation of the results. Characteristic absorbance bands were identified for each sample, allowing for their assignment to functional groups of the main components of the composite matrix, such as glass fibers, polyester resins, and epoxy resins (Figure 2). WT1 was identified as PET: the band at 1713 cm^−1^ is associated with stretching vibrations of carbonyl groups (C=O) in esters, 1241 cm^−1^ and 1097 cm^−1^ correspond to C-O stretching vibrations in ester bonds, whereas 722 cm^−1^ is a characteristic band corresponding to C-H bending vibrations in methyl chains, which is also typical for PET [43,44]. The WT2 spectrum shows bands characteristic of epoxy resins, inferred from the presence of the band around 915 cm^−1^ typical for epoxy groups (C-O-C) [45], bands around 1735 cm^−1^ suggesting the presence of carbonyl groups (C=O), often found in polyester resins or other additives, and bands in the range of 1250–1150 cm^−1^ related to C-O bond vibrations [46]. The WT5 spectrum is very similar to WT2, indicating the presence of epoxy resins, but with some additional signals that may suggest the presence of polyamides. Bands in the range of 3400–3300 cm^−1^ may originate from N-H stretching vibrations, and bands at 1640 cm^−1^ correspond to stretching vibrations of amide groups (-(C=O)N=), which may also suggest the presence of polyamide [47]. As in the previous samples, the WT10 spectrum indicates the presence of polyester or epoxy resins, which can be inferred from the band around 1730 cm^−1^, 1236 cm^−1^, and 1100–1000 cm^−1^. The WT3 spectrum is typical for a straight hydrocarbon chain polymer: the band around 2916 cm^−1^ and 2849 cm^−1^, characteristic of the symmetric and asymmetric vibrations of CH_3_ and CH_2_, and 1462 cm^−1^ and 1377 cm^−1^ deformational vibrations of the CH_2_ group (for PE) and CH_3_ (for PP) [15]. In the WT4 spectrum, the broad band around 3500–3300 cm^−1^ may suggest the presence of hydroxyl groups (-OH), which may indicate a small amount of impurities or compounds such as polyamides. On the other hand, the band around 1716 cm^−1^ may correspond to carbonyl vibrations (C=O), characteristic of ester or amide groups, suggesting the possible presence of polyamide (PA) or polyester resin. Microscopic images of WT6 (Table 2) suggest wood. The broad band 3400–3300 cm^−1^ in the FTIR spectrum may correspond to -OH groups, present in natural compounds such as cellulose [48]. The band in the range of 1724 cm^−1^ is typical for carbonyl groups (C=O), which may be related to the presence of components in wood or epoxy matrix. Signals 1600–1500 cm^−1^ may correspond to aromatic or deformational vibrations of C=C, which is consistent with the presence of epoxy resin. Furthermore, 1026 cm^−1^ and 1061 cm^−1^ are assigned to vibrations of C-O-C and C-O groups, characteristic of cellulose and epoxy resins. The spectra for WT8 and WT11 are quite similar and, due to the presence of characteristic bands for carbonyl groups, C-O and C-H indicate a polyester resin as the probable component. Based on the signals in the WT10 and WT12 spectrums, it is concluded that the samples contain a polyester resin or other substance based on an ester polymer with aromatic rings.

#### 3.1.3. Thermogravimetric Analysis (TGA)

**Figure 3:**
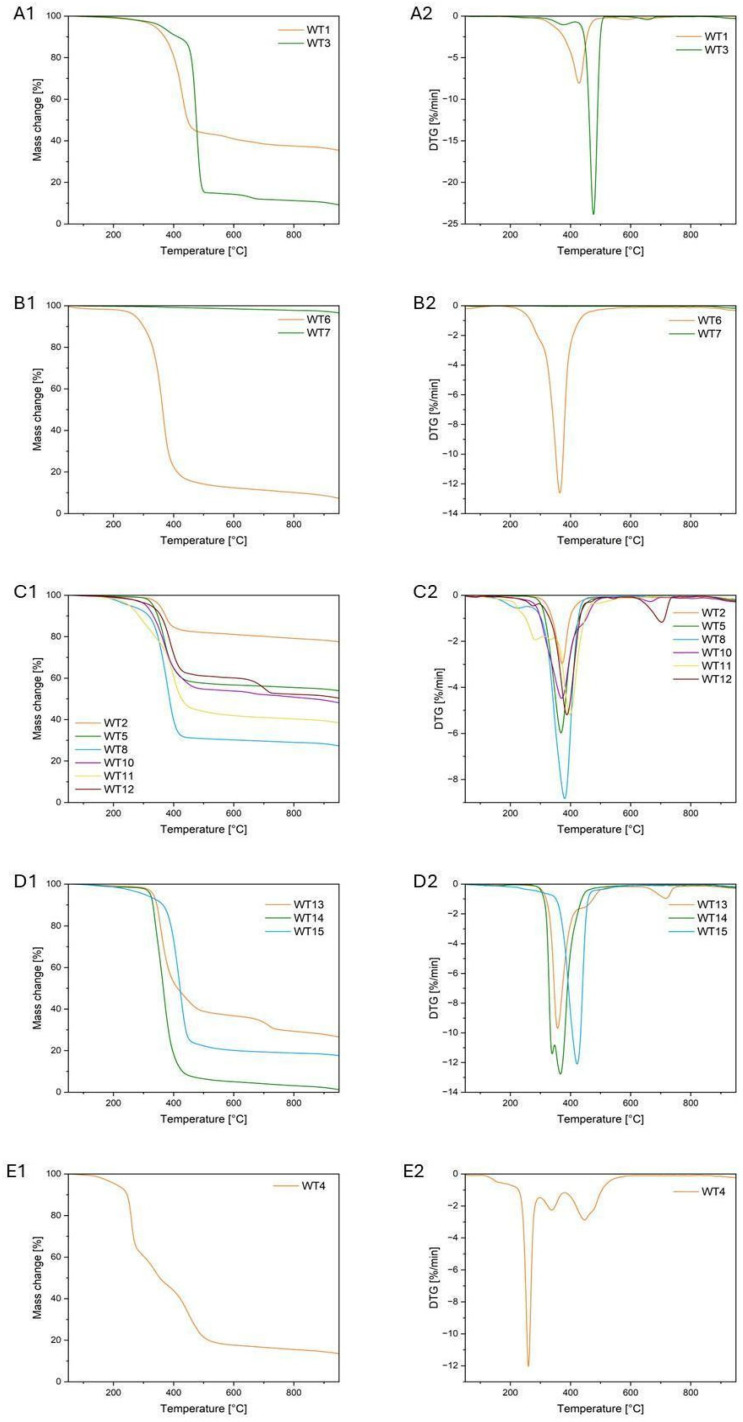
TGA curves of (**A1**) WT1, WT3; (**B1**) WT6, WT7; (**C1**) WT2, WT5, WT8, WT10-12; (**D1**) WT13-WT15; (**E1**) WT4 and DTG curves of (**A2**) WT1, WT3; (**B2**) WT6, WT7; (**C2**) WT2, WT5, WT8, WT10-12; (**D2**) WT13-WT15; (**E2**) WT4 in nitrogen atmosphere.

The process of the thermal decomposition of the samples was carried out in a nitrogen atmosphere (Figure 3). The determined parameters, including temperatures of 1% and 5% mass loss, the temperature of the start of degradation, the temperature of the maximum rate of mass loss, and the residual mass are summarized in (Table 4). Thermogravimetric analysis (TGA) confirms the complexity of materials used in wind turbines. The residual masses after pyrolysis range from 1.03% to 96.37%, indicating the presence of materials with diverse thermal properties. This wide range of residual values suggests the presence of both organic substances, which undergo complete degradation, and inorganic materials with significantly higher thermal stability. For the WT1 sample (PET), the main decomposition stage occurs between 290 °C and approximately 480 °C, with the maximum degradation rate observed at 428.7 °C, which aligns with data from the literature [49]. An additional peak of low intensity (T_max2_ = 589.6 °C) is observed, with a residual mass of approximately 35%, which may indicate the presence of additives or fillers such as glass fibers. The TGA and DTG curves for sample WT3 exhibit a characteristic pattern typical of thermoplastic polymers, as confirmed by FTIR analysis. The maximum degradation rate for WT3, at 476.6 °C, aligns with data from the literature for polyethylene (PE). Figure 3 presents the mass loss curve and DTG derivative for sample WT6, which was identified as wood based on microscopic and spectroscopic analyses (Section 3.1.2 and Section 3.1.3). Sample WT6 shows a residual mass of 6.91%, with the maximum mass loss rate occurring at 365.0 °C. During the pyrolysis process, the wood sample exhibits maximum mass loss at temperatures below 500 °C, characteristic of cellulose decomposition [50]. Sample WT7, being a metal, exhibits the highest residual mass, amounting to 96.7%. The samples WT2, WT5, WT8, and WT10 show similar TGA curves, with differences observed in their residual mass (27–77%). The temperatures of maximum degradation rate are within a similar range. FTIR analysis indicates that these materials are resin-based, commonly used in wind turbine blades, and the differences in residual mass may be attributed to varying amounts of glass fibers. The TGA and DTG curves for sample WT4 suggest a three-stage degradation process, indicating a complex structure whose characteristics cannot be fully defined based solely on thermogravimetric analysis. Samples WT12 and WT13 exhibit a similar thermal degradation profile, characterized by two distinct peaks, suggesting a two-stage degradation process. In the first stage, at a maximum degradation rate temperature (T_max1_) of 360–380 °C, organic matter and resin components undergo degradation. The second stage, where T_max2_ reaches 704–717 °C, indicates the presence of components stable at high temperatures, such as reinforcing fibers or fillers. The presence of two distinct thermal degradation stages confirms the heterogeneous nature of samples WT12 and WT13, which consist of both organic components (resin) and inorganic additives. Samples WT14 and WT15 exhibit a one-step degradation process, with a T_max_ range of 360–420 °C. The difference in residual mass is due to the varying content of inorganic additives.

#### 3.1.4. Differential Scanning Calorimetry (DSC)

**Figure 4:**
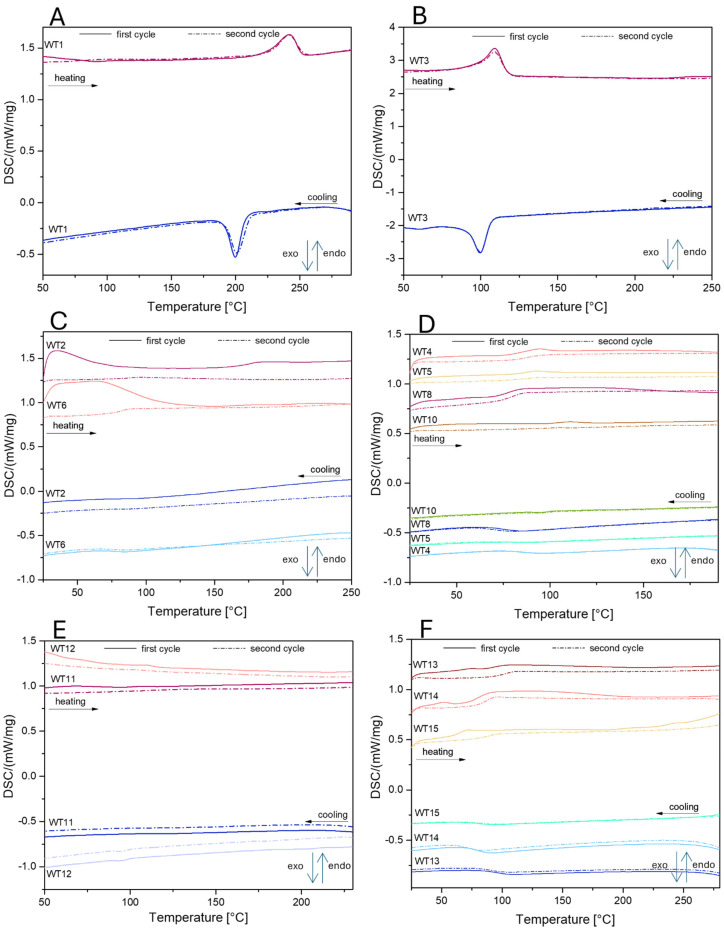
DSC curves of (**A**) WT1; (**B**) WT3; (**C**) WT2, WT6; (**D**) WT4-5, WT8, WT10; (**E**) WT11-12; (**F**) WT13-15.

For more detailed material characterization and to confirm the results of the FTIR and TGA analyses, differential scanning calorimetry (DSC) was also performed (Figure 4). This method allowed for the identification of characteristic phase transitions in the polymer samples, such as the glass transition temperature (T_g_), crystallization temperature (T_c_), and melting temperature (T_m_). The DSC curve of the WT1 sample exhibits characteristic endothermic and exothermic effects associated with phase transitions typical for PET. In both the first and second heating cycles, a distinct endothermic peak appears around 243 °C, corresponding to the melting process of PET. During the cooling cycles, an exothermic peak is observed at approximately 200 °C, associated with PET’s crystallization process. The presence of this exothermic crystallization effect indicates the sample’s ability to partially reorganize its molecular structure after melting, a behavior typical of semi-crystalline polymers. The observed melting and crystallization temperatures align with PET values in the literature, confirming the material’s identification as polyethylene terephthalate [51]. WT3, identified as polyethylene based on FTIR analysis, exhibits two characteristic peaks: an endothermic peak in the heating cycle at 112.5 °C and an exothermic peak in the cooling cycle at 100.1 °C. According to the literature, the first peak corresponds to the melting temperature of polyethylene, while the second represents the crystallization temperature. These thermal transitions align with the well-documented behavior of polyethylene, reinforcing the conclusions drawn from the FTIR analysis [52]. For samples WT10, WT11, and WT12, no phase transitions are observed in either the heating or cooling cycles during DSC analysis. Based on previous analyses, these samples were identified as polyester resin with a high fiber content (high residual mass confirmed by TGA, Table 4). The presence of a significant amount of fibers likely affects the thermal behavior of these samples, reducing the likelihood of observable phase transitions typically associated with the polymer matrix. In the case of samples WT4–WT6, WT8, WT10, and WT13–WT15 low-intensity peaks are observed in the heating cycles within the temperature range of 83 to 110 °C. These minor transitions may indicate the glass transition temperature (T_g_) typical for epoxy resins. The temperature differences may be due to the different compositions of these systems (including different additive and fiber contents). Although weak, these thermal effects suggest the presence of an amorphous structure in the epoxy, where molecular rearrangements begin around T_g_, causing slight enthalpic changes. This observation aligns with the behavior of epoxy resins, which are known for their low-intensity T_g_ peaks due to the limited mobility in their crosslinked networks.

### 3.2. Materials After Processing

#### 3.2.1. Fraction Analysis

**Figure 5:**
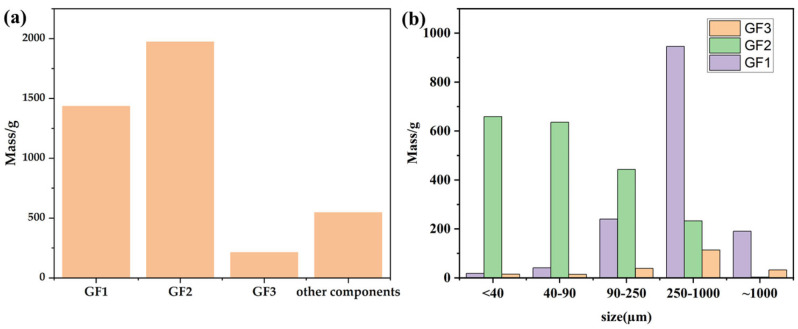
Mass sample of different fractions: (**a**) summed up for GF1-3 and others; (**b**) based on the size.

Based on the wet separation process, three fractions—GF1, GF2, and GF3—along with other components were obtained, as detailed in Table 5, Figure 5. The total masses of GF1, GF2, and GF3 were 1434.5 g, 1972.5 g, and 212.5 g, respectively. GF2 represents the largest fraction by mass, likely due to its higher density, with the particle size predominantly distributed within the 40–90 µm (635.5 g) and <40 µm (658.5 g) ranges. In contrast, GF3 has the smallest mass and density, with most particles concentrated in the 250–1000 µm (113.5 g) and <40 µm (14.5 g) ranges. Due to its lower density, GF3 floats on the water surface during separation. Additionally, other components were separated with a total mass of 546 g. This distribution highlights the variability in density and particle size across fractions, significantly influencing the separation outcomes.

#### 3.2.2. Optical Microscopy

Table 6 presents a series of microscopic images capturing the morphology of recycled materials containing glass fiber (GF) divided into distinct size fractions through sieving. These fractions were separated based on particle size, and each sample is presented for three different GF materials (GF1, GF2, and GF3) across various size ranges. The images provide a detailed visual comparison of the physical structure of the glass fibers, highlighting differences in particle shape, size, and material composition across the fractions. In the fractions greater than 250 µm, the images reveal the presence of large, compacted fragments of glass fibers. These fragments exhibit a relatively bulky structure, with fibers appearing densely packed or clustered. Additionally, in the GF3 samples, other materials can be observed, which are identified in the next section through thermogravimetric measurements as organic matter. As the sieve size decreases and finer fractions are observed (e.g., the 90–250 µm and smaller), the material becomes progressively more fragmented. These finer fractions contain a significant proportion of elongated fibers, whose shapes are governed by the intrinsic geometry of the glass fibers themselves. The elongated, thread-like appearance of these fibers is particularly evident in the smallest particle ranges (40–90 µm and <40 µm), where the majority of the material consists of thin, slender fibers.

#### 3.2.3. Thermogravimetric Analysis (TGA)

**Figure 6:**
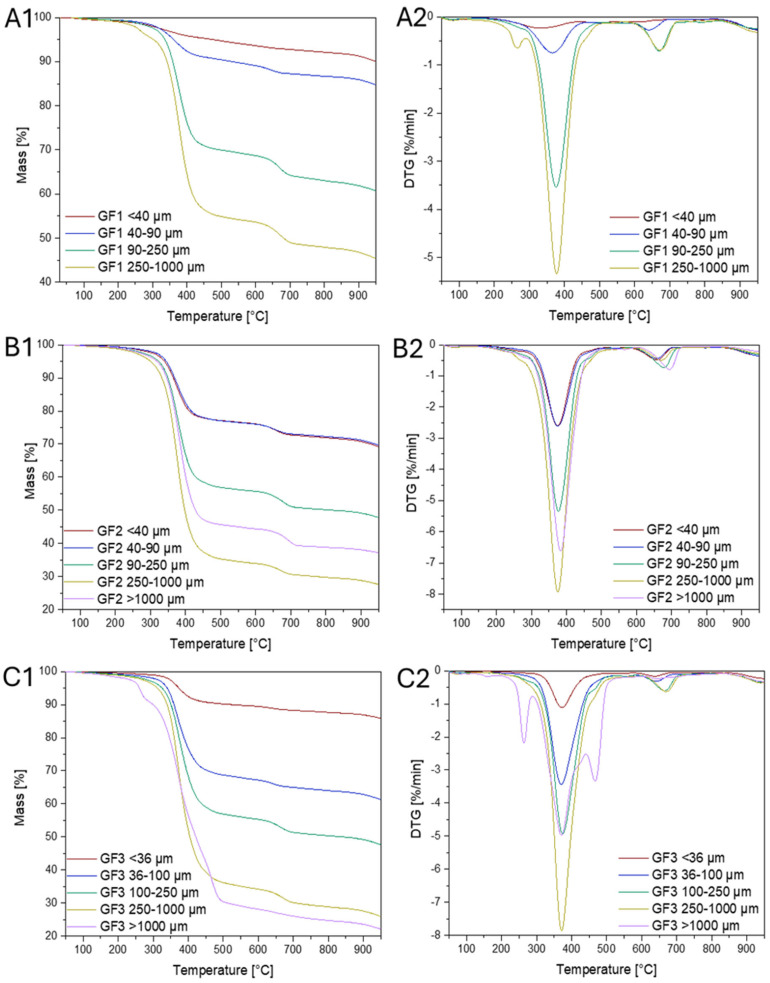
TGA (**A1**–**C1**) and DTG (**A2**–**C2**) curves of glass fibers in nitrogen atmosphere.

The thermogravimetric analysis (TGA) was conducted in a nitrogen atmosphere for materials containing glass fibers derived from ground wind turbine materials, which were sieved into different particle size fractions (Figure 6). The results of these studies demonstrate significant differences in the thermal decomposition behavior depending on particle size. Analysis of the TGA curves revealed that as the samples size increases, the decomposition process begins at lower temperatures, as observed, for example, based on the temperature at 5% mass loss (Table 7). All samples exhibit multi-stage decomposition (ranging from 2 to 4 stages), indicating the diverse materials present in wind turbines, which consist of both polymers and fillers. The derivative thermogravimetric (DTG) curves analysis showed distinct decomposition peaks in the temperature range of 300–400 °C, which can be attributed to the decomposition of organic polymers, such as epoxy resins used in wind turbine production. The decomposition rate in this temperature range suggests intensive degradation of the polymer matrix, which constitutes the primary structural component of these materials. Subsequent decomposition stages, occurring at higher temperatures (above 400 °C), may indicate the decomposition of more thermally stable components, which are characterized by greater heat resistance. These findings align with the thermal behavior of polymer composites, where the decomposition of individual components occurs over a wide temperature range, reflecting the diverse physicochemical properties of these materials. Additionally, noticeable differences in pyrolytic residue were observed, which depend on fiber size. Larger fiber sizes exhibit lower residual mass after decomposition, suggesting a higher organic (resin) content in the samples. In contrast, smaller fibers show a greater amount of residue, which can be attributed to a higher proportion of inorganic materials, such as glass fibers or other composite reinforcements. The sample with the highest residual mass was GF1, while GF3 showed the lowest residue. These differences can be explained based on separation methods. When water is added to the mixture, GF3 floats on the surface, while GF1 and GF2 sink. This suggests that GF1 may contain a higher proportion of larger inorganic materials, such as glass fibers, which are more stable during decomposition in high temperatures. As shown in Table 5, the main sizes of GF1 range from 250 to 1000 µm, and even larger than 1000 µm, leading to faster aggregation when water is added. GF2 consists of smaller inorganic particles, which makes the separation process slower. Conversely, GF3 likely consists of a larger proportion of organic materials, which decompose more completely, leaving less residue, which explains its ability to float on the water’s surface. Currently known methods for the mechanical recycling of fiber-reinforced polymers provide epoxy powder, individual carbon fibers, and CFRP particles [53]. However, the inclusion of additional wet and dry separation stages, as presented in this study, allows for the production of fractions characterized by a higher fiber content compared to organic matter. For instance, in the case of the GF1 < 40 µm fraction, thermogravimetric analysis (TGA) revealed that the residual mass is as high as 89.7%, indicating a predominance of glass fibers. This result highlights the effectiveness of the proposed methods in facilitating the efficient recovery of high-value materials.

## 4. Conclusions

This work presents the comprehensive composition of materials derived from turbine blades, highlighting wind turbine waste’s diverse and complex nature. By employing straightforward instrumental analysis techniques such as TGA, DSC, FTIR, and microscopy, it was possible to qualitatively identify key components, including thermoplastic polymers (PET, PE, and PP), epoxy and polyester resins, wood, metals, and fillers such as glass fibers. This study demonstrates the highly complex and heterogeneous composition of waste derived from the recycling of wind turbines.

For the first time, an innovative approach to recycling decommissioned wind turbines was implemented, introducing a novel compressive milling process as an efficient alternative to traditional knife mills, which reduced tool wear. The fractionation of materials, conducted using both wet and dry separation techniques, enabled the isolation of distinct material fractions (GF1, GF2, and GF3) with different compositions and thermal properties. Thermal analyses revealed that GF1 had a high residual mass (89.7%), indicating a predominance of stable materials such as glass fibers, while GF3 contained more organic content, reflected in its lower thermal stability. This result highlights the effectiveness of the proposed methods in facilitating the efficient recovery of high-value materials. Moreover, this approach offers significant environmental benefits. The process minimizes particulate emissions by employing compressive milling and combining wet and dry separation techniques. The innovative methodology enhances material recovery while maintaining environmental and economic sustainability.

This approach represents a significant advancement in wind turbine recycling, addressing the challenging waste stream generated after their decommissioning. Simultaneously, it paves the way for the efficient recovery of valuable components, which is crucial for advancing circular economy practices. Future research should aim to further optimize these techniques and investigate the economic feasibility of scaling the process.

## Figures and Tables

**Table 1:** Components of wind turbines.

Part of Turbines	Materials	Percent	Reference
foundation	concrete	80–90%	[23]
	steel	10–20%	[20,21,22]
tower	steel	95–98%	[24,25,26]
	others (eg.wood, concrete)	2–5%	
nacelle/gearbox/generator	steel and various alloys composites and hybrids lubricants earth based permanent magnets copper	50–60%	[27,28]
		10–20%	
		5–10%	
		10–15%	
hub	steel aluminium, iron	80–90%	[28,29]
		10–20%	
blades	glass fiber, carbon fibres, wood laminates polyester resins, epoxies steel and other materials	70-80%	[14,21,30]
		5–10%	
		15–25%	
		5–10%	
electronical and control system	copper	60–70%	[31,32]
	silicon	20–30%	
cables and busbars	plastic	10–20%	[31,33]
	copper	40–50%	
	aluminium	30–40%	
miscellaneous	lubricants, grease paint rubber, plastic	40–50%	[31,34,35]
		10–20%	
		30–40%	

**Table 2:** Sample labels and corresponding photos.

Label	Photo	Label	Photo
WT1	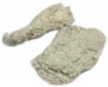	WT8	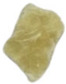
WT2	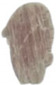	WT9	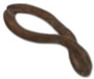
WT3	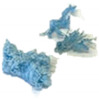	WT10	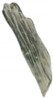
WT4	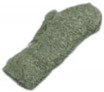	WT11	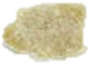
WT5	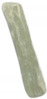	WT12	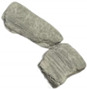
WT6	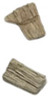	WT13	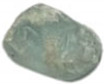
WT7	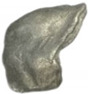	WT14	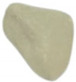
WT15	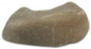		

**Table 3:** Microscopic images of samples WT1–WT15.

Label	Photos		
	**mag. ×100**	**mag. ×200**	**mag. ×500**
WT1	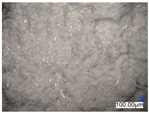	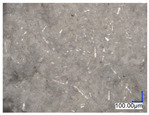	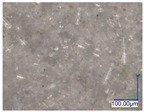
WT2	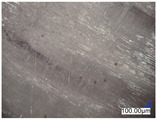	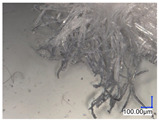	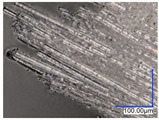
WT3	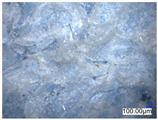	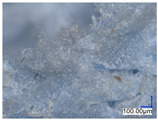	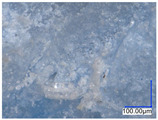
WT4	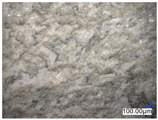	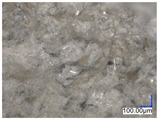	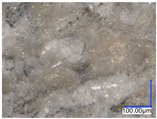
WT5	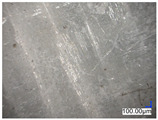	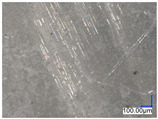	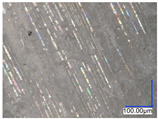
WT6	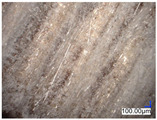	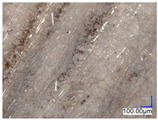	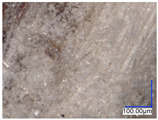
WT7	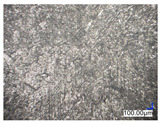	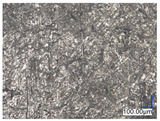	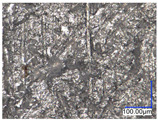
WT8	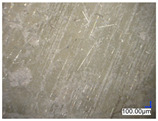	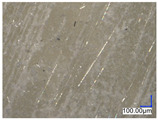	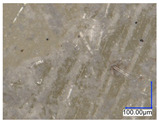
WT9	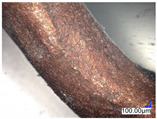	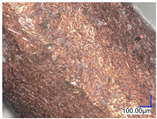	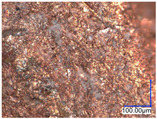
WT10	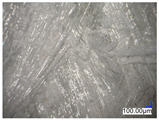	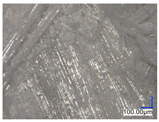	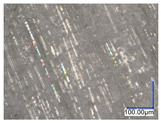
WT11	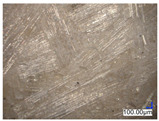	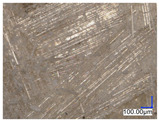	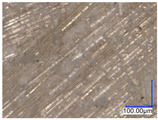
WT12	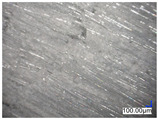	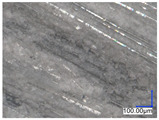	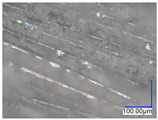
WT13	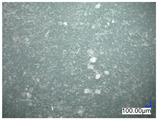	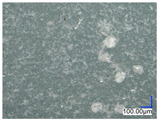	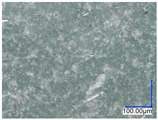
WT14	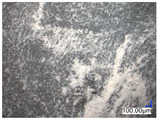	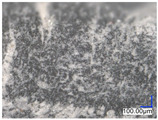	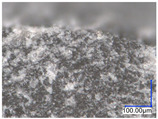
WT15	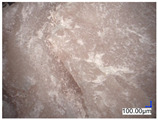	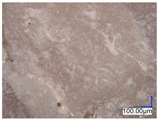	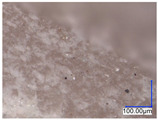

**Table 4:** Results of thermal analysis.

Code	Temperature at 1% Weight Loss/°C	Temperature at 5% Weight Loss/°C	Temperature at Maximum Weight Change Rate/°C	Residual Mass/%
WT1	207.9	337.2	428.7/589.6	35.17
WT2	277.8	354.0	372.0	77.18
WT3	228.4	360.0	476.6	8.70
WT4	137.0	207.9	260.4/336.7/447.8	13.24
WT5	288.2	332.2	368.5	53.71
WT6	75.2	273.1	365.0	6.91
WT7	-	-	80.6	96.37
WT8	167.7	254.8	225.6/380.9	27.12
WT9 **	-	-	-	-
WT10	188.9	309.2	370.7/665.6	47.81
WT11	164.3	253.8	283.7/398.8	38.18
WT12	210.0	324.5	275.7/388.5/704.0	49.96
WT13	236.8	332.8	356.7/717.0	26.04
WT14	165.1	323.7	366.2	1.03
WT15	176.5	304.1	422.0	17.40

** metal; unable to measure.

**Table 5:** The mass of all components.

	Mass/g			
	GF1	GF2	GF3	Other Components
>1000 µm	190	3	32	-
250–1000 µm	945.5	232.5	113.5	-
90–250 µm	240	443	38.5	-
40–90 µm	41	635.5	14	-
<40 µm	18	658.5	14.5	-
sum	1434.5	1972.5	212.5	546

**Table 6:** Microscopic images of samples GF1-3 with different sizes.

Label		Photos	
	GF1	GF2	GF3
>1000 µm	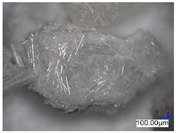	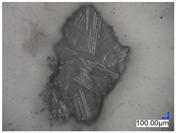	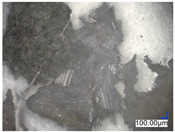
250–1000 µm	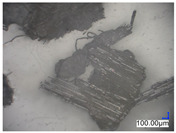	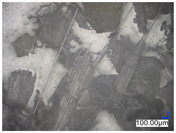	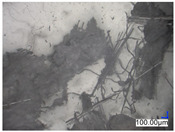
90–250 µm	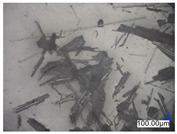	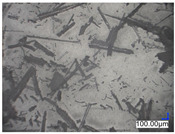	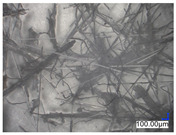
40–90 µm	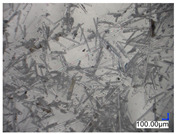	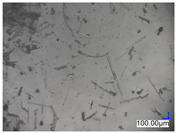	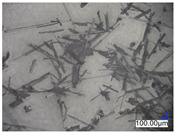
<40 µm	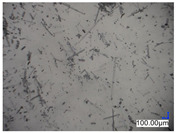	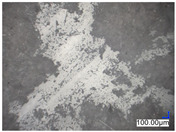	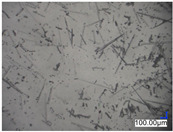

**Table 7:** Results of thermal analysis.

Code	Temperature at 1% Weight Loss/°C	Temperature at 5% Weight Loss/°C	Temperature at Maximum Weight Change Rate 1/°C	Temperature at Maximum Weight Change Rate 2/°C	Residual Mass/%
GF1 < 40 µm	224.7	475.3	330.9	599.8	89.7
GF1 40–90 µm	254.0	361.4	366.2	640.3	84.4
GF1 90–250 µm	238.7	336.2	375.9	669.6	60.4
GF1 250–1000 µm	196.5	303.0	267.2/377.1	670.3	44.9
GF2 < 40 µm	236.1	389.7	374.4	657.6	68.8
GF2 40–90 µm	240.1	344.8	375.3	655.9	69.2
GF2 90–250 µm	209.4	323.2	377.2	676.6	47.4
GF2 250–1000 µm	188.8	300.4	375.8	671.5	27.3
GF2 > 1000 µm	209.1	318.5	383.4	693.8	36.9
GF3 < 36 µm	292.0	373.5	372.0	636.4	85.6
GF3 36–100 µm	226.2	339.9	370.8	640.8	60.8
GF3 100–250 µm	206.4	325.4	374.6	664.3	47.3
GF3 250–1000 µm	179.7	311.9	371.7	669.8	25.5
GF3 > 1000 µm	153.2	260.5	263.8/371.7	467.1	21.8

## Data Availability

The original contributions presented in the study are included in the article, further inquiries can be directed to the corresponding author.
